# Large-Scale Isolation Facilities and Potential for Secondary Infectious Disease Outbreak

**DOI:** 10.3201/eid2701.203127

**Published:** 2021-01

**Authors:** Shi Yu D. Lim, Hong Liang Tey

**Affiliations:** National Skin Centre, Singapore (S.Y.D. Lim, H.L. Tey);; Woodlands Health Campus, Singapore (S.Y.D. Lim, H.L. Tey);; National University of Singapore (H.L. Tey);; Nanyang Technological University, Singapore (H.L. Tey)

**Keywords:** chickenpox, coronavirus disease, infections, COVID-19, influenza, SARS-CoV-2, severe acute respiratory syndrome coronavirus 2, viruses, respiratory infections, varicella

**To the Editor:** Singapore has instituted large-scale isolation facilities similar to those detailed by Choi et al. (*1*) for patients with mild coronavirus disease. We highlight the risk for transmission of secondary infectious diseases by sharing our experience with a varicella outbreak.

Three patients, all migrant workers housed in the same isolation hall, were seen for vesicular eruptions, later laboratory confirmed as varicella, within the span of 9 days. The first patient’s symptoms were truncal erythematous-based vesicles and erosions after a prodrome of fever and headache. He was promptly transferred for further hospital isolation. As part of a ring vaccination strategy, we offered 200 close contacts postexposure vaccination. However, 2 other patients, not close contacts of the first, had similar eruptions; for the second patient, 7 days later with a rash duration of 2 days, and for the third, 8 days after, with a rash duration of 6 days (Figure). After these additional cases, vaccination was offered to all remaining patients in the isolation facility. 

**Figure:**
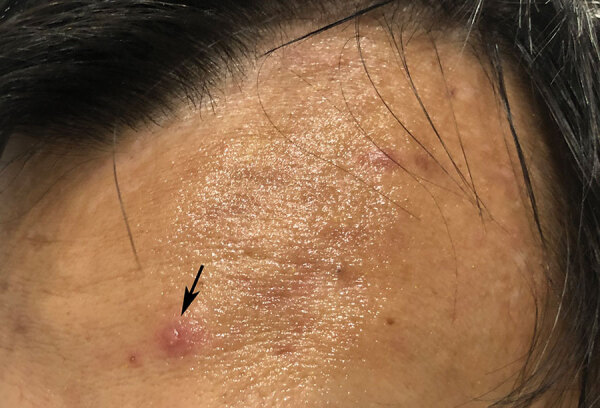
Vesicle on an erythematous base (arrowed), commonly described as “dewdrop on rose petal”, over the forehead of a patient with varicella, Singapore, 2020.

All 3 patients probably contracted varicella from unidentified persons with varicella or zoster infection, given that illness onset fell short of the usual 10–21-day incubation period (*2*). Although varicella seroprevalence among adults in Singapore is high (88%), data on seroprevalence among migrant workers remain limited (*3*).

Although isolation facilities obviate the capacity constraints of hospital isolation, our experience highlights the potential for secondary outbreaks, which are disruptive and costly to investigate and control. To mitigate this risk, preentry screening inquiring about previous chickenpox infection or vaccination should be considered. Serologic screening is ideal but challenging to implement. Among patients, social distancing and face coverings should be enforced. We also recommend active surveillance for vesicular rash and fever, prompt isolation of patients with suspected cases, and vaccination of identified close contacts without previous infection, vaccination, or contraindications to vaccination, as well as temporarily halting patient flow while these measures are implemented.
